# Soil bacterial community as impacted by addition of rice straw and biochar

**DOI:** 10.1038/s41598-021-99001-9

**Published:** 2021-11-12

**Authors:** Zhiqiang Tang, Liying Zhang, Na He, Diankai Gong, Hong Gao, Zuobin Ma, Liang Fu, Mingzhu Zhao, Hui Wang, Changhua Wang, Wenjing Zheng, Wenzhong Zhang

**Affiliations:** 1 Shenyang Agricultural University, Shenyang, China; 2 Liaoning Rice Research Institute, Shenyang, China

**Keywords:** Microbiology, Molecular biology, Environmental sciences

## Abstract

The application of straw and biochar can effectively improve soil quality, but whether such application impacts paddy soil bacterial community development remains to be clarified. Herein, the impacts of three different field amendment strategies were assessed including control (CK) treatment, rice straw (RS) application (9000 kg ha^−1^), and biochar (BC) application (3150 kg ha^−1^). Soil samples were collected at five different stages of rice growth, and the bacterial communities therein were characterized via high-throughput 16S rDNA sequencing. The results of these analyses revealed that soil bacterial communities were dominated by three microbial groups (*Chloroflexi, Proteobacteria* and *Acidobacteria*). Compared with the CK samples, *Chloroflexi, Actinobacteria, Nitrospirae* and *Gemmatimonadetes* levels were dominated phyla in the RS treatment, and *Acidobacteria, Actinobacteria, Nitrospirae* and *Patescibacteria* were dominated phyla in the BC treatment. Compared with the RS samples, *Chloroflexi*, *Acidobacteria*, *Actinobacteria*, and *Verrucomicrobia* levels were increased, however, *Proteobacteria*, *Gemmatimonadetes*, *Nitrospirae*, and *Firmicute* levels were decreased in the BC samples. Rhizosphere soil bacterial diversity rose significantly following RS and BC amendment, and principal component analyses confirmed that there were significant differences in soil bacterial community composition among treatment groups when comparing all stages of rice growth other than the ripening stage. Relative to the CK treatment, *Gemmatimonadaceae, Sphingomonadaceae, Thiovulaceae, Burkholderiaceae,* and *Clostridiaceae-1* families were dominant following the RS application, while *Thiovulaceae* and *uncultured-bacterium-o-C0119* were dominant following the BC application. These findings suggest that RS and BC application can improve microbial diversity and richness in paddy rice soil in Northeast China.

## Introduction

Paddy rice is a key agricultural crop in China, with japonica rice primarily being cultivated in Northeast China. After harvesting, farmers traditionally burn the rice straw that remains after harvesting before planting rice the following spring. However, air pollution concerns have led to the prohibition of such crop residue burning practices. Instead, agricultural residues are applied to fields in an effort to minimize environmental harm while maximizing resource utilization^1^.

Many researchers have advocated the application of straw and biochar to fields in an effort to improve soil quality and nutrient availability, providing key substrates that can support the growth of root-associated microbes and enzymatic activity^2,3^. Biomass represents a renewable source of fixed carbon (C) that can directly impact soil bacterial community composition and richness relatively quickly^4^. Applying agricultural residues such as rice, wheat, and maize straw can thus significantly improve the C supply available in a given cropping system.

Biochar (BC) is a solid product derived from the pyrolysis or gasification of crop residues at a low temperature (< 500 °C) under anoxic conditions^5^. BC is considered a viable soil amendment compatible with sustainable agricultural practices, and it can significantly improve nutrient availability, soil health, and soil productivity in agricultural contexts^6^. By improving soil water retention and cationic adsorption, BC can also decrease nutrient loss and improve the quality of acidic soils. BC application can further slow the release of C and N owing to changes in soil properties^7^, thereby influencing microbial community composition and activity^8^. As BC is porous, it can also serve as an environmental niche compatible with sheltered growth in the soil^9^. Applying BC can thus profoundly alter soil physicochemical properties, thereby impacting microbial richness and diversity. However, the mechanisms whereby BC alters soil biological properties remain to be fully clarified^10^.

Effectively characterizing soil microbial communities is challenging, but recent advances in high-throughput sequencing technologies have significantly aided these efforts^11^. Microbes within the soil can associate with the roots of plants, and modulate crop growth, nutrient uptake, and disease susceptibility^12^. While studies of these microbial communities associated with rice, wheat, and maize plants have shown that both soil type and host genotype influence community composition^13,14^, how these communities respond to BC or straw application remains to be clearly defined. In some studies, soil microbial community composition has been found not to vary when comparing fertilized and non-fertilized soils in a long-term winter wheat, although other studies have instead observed changes in these communities following the application of crop straw or other organic/inorganic fertilizers. These inconsistent results may be a result of differences in environmental or other management strategies among studies^15–17^.

This study was designed to assess soil bacterial community responses to the application of rice straw (RS) and BC via a high-throughput 16S rDNA sequencing approach. For these analyses, we tracked rhizosphere microbiome development from the elongation to the ripening stage of rice growth in field trials, and we used a Random Forest model to explore the relationships between rhizosphere microbiota composition and rice straw or biochar application.

## Results

### Assessment of rice rhizosphere soil bacterial community and richness

**Figure 1:**
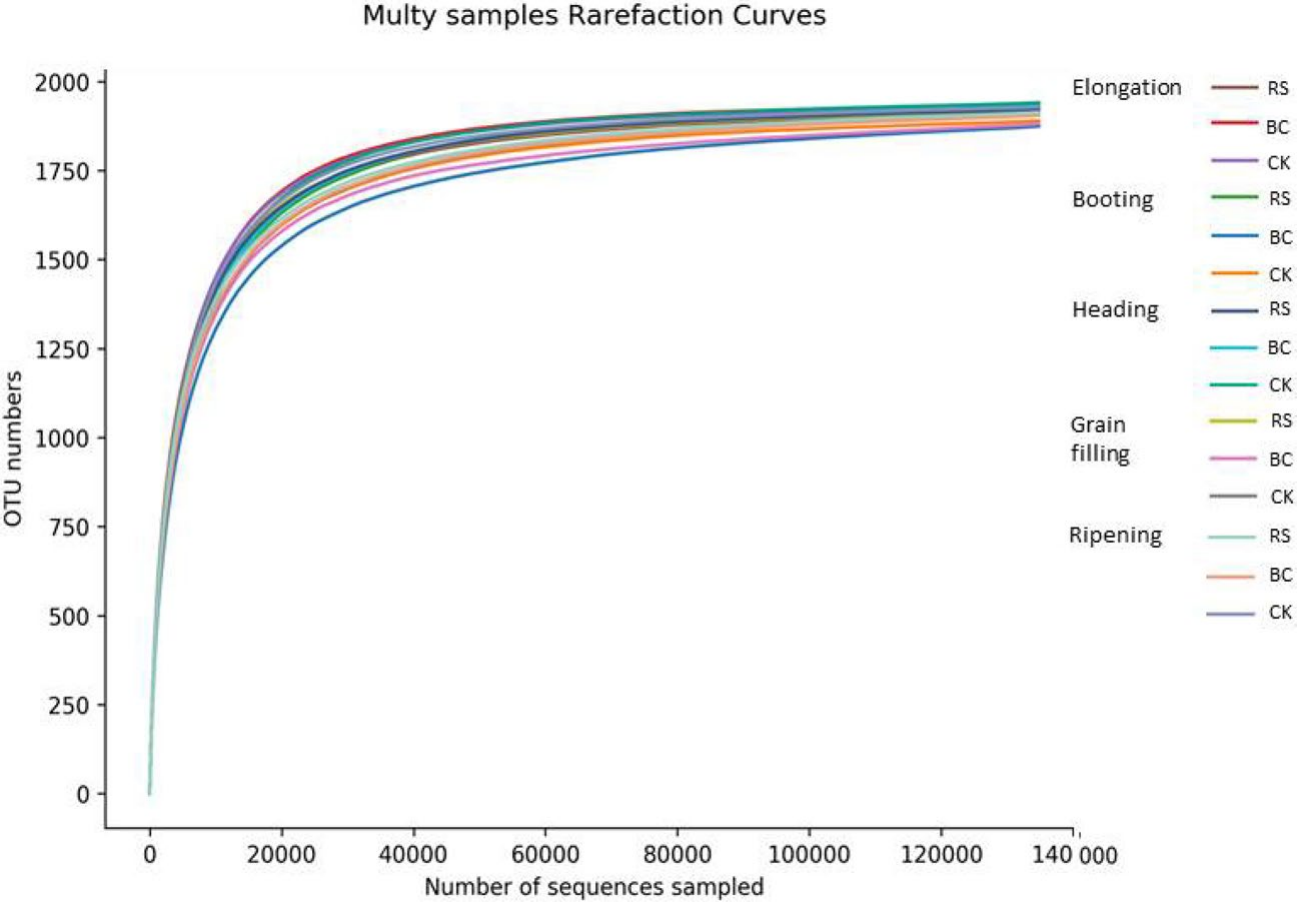
Rarefaction on species-abundance data. Treatments: *RS* rice straw applied at 9000 kg ha^−1^, *BC* biochar applied at 3150 kg ha^−1^, *CK* soil without straw/biochar application.

In total, over 40,000 valid clean reads were obtained per treatment via a sequence optimization process, after which soil microbial community richness (OTU number) index values were calculated (Table 1). No significant differences in OTUs were observed when comparing samples in different groups (Fig. 1). CK treatment was associated with lower Chao1 index values relative to the RS treatment, suggesting that the RS amendment improved bacterial community diversity at the booting stage of rice growth, although community composition tended to stabilize after this stage. Shannon index values in the RS treated samples exhibited improved community diversity relative to samples in the BC or CK treatment groups.

**Table 1:** Estimated operational taxonomic unit (OTU) richness and diversity indexes for soil samples from different stages of rice growth following 16S rDNA gene library clustering at 97% identity after Illumina Hiseq (2500) sequencing.

Stage	Treatment	Observed OTUs	Chao1	Shannon
Elongation	RS	1918	1952.2	9.64
	BC	1937	1948.1	9.52
	CK	1936	1951.0	9.58
booting	RS	1920	1933.6	9.30
	BC	1875	1920.5	9.13
	CK	1890	1909.5	9.37
heading	RS	1923	1947.8	9.43
	BC	1933	1950.9	9.33
	CK	1941	1967.0	9.34
grain filling	RS	1923	1944.6	9.50
	BC	1882	1945.5	9.32
	CK	1906	1936.6	9.39
ripening	RS	1914	1939.0	9.50
	BC	1905	1937.1	9.39
	CK	1929	1943.9	9.50

Treatments: *RS* rice straw applied at 9000 kg ha^−1^, *BC* biochar applied at 3150 kg ha^−1^, *CK* soil without straw/biochar application.

### Bacterial community composition

**Figure 2:**
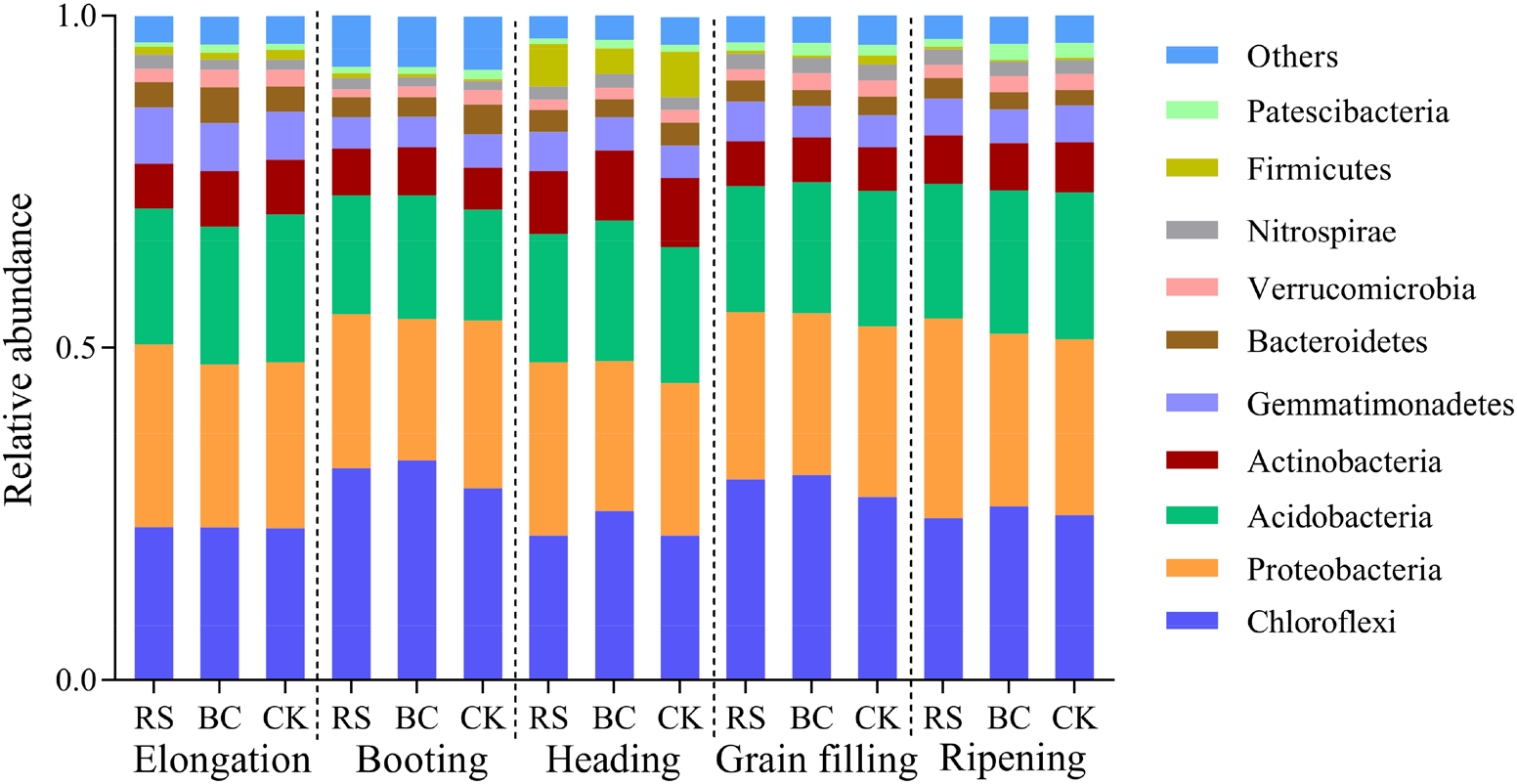
Phylum level bacterial community composition following the rhizosphere treatments. Treatments: *RS* rice straw applied at 9000 kg ha^−1^, *BC* biochar applied at 3150 kg ha^−1^, *CK* soil without straw/biochar application.

Rhizosphere bacterial community composition during different stages of growth and under different treatment conditions was next assessed (Fig. 2). In comparison with the CK, RS and BC amendment did not alter the top 10 phyla present within the sequenced bacterial community, although significant diversity was observed with respect to the bacteria within each phylum at all five growth stages. The three most dominant phyla in these samples were *Chloroflexi, Proteobacteria,* and *Acidobacteria*, with these sequences accounting for > 60% of total sequences in all soil samples.

*Chloroflexi, Actinobacteria,* and *Gemmatimonadetes* levels were higher in RS samples relative to CK samples from the booting stage to the grain filling stage, while *Nitrospirae* levels were significantly higher relative to CK treatment in all RS-treated samples other than the grain-filling stage samples. RS amendment was associated with a significant increase in *Proteobacteria* content relative to CK treatment at the ripening stage. BC application was associated with increased *Chloroflexi* abundance during all growth stages relative to CK treatment. Abundance of *Acidobacteria, Actinobacteria, Nitrospirae,* and *Patescibacteria* increased in BC samples relative to CK samples from the booting stage to the grain filling stage.

**Figure 3:**
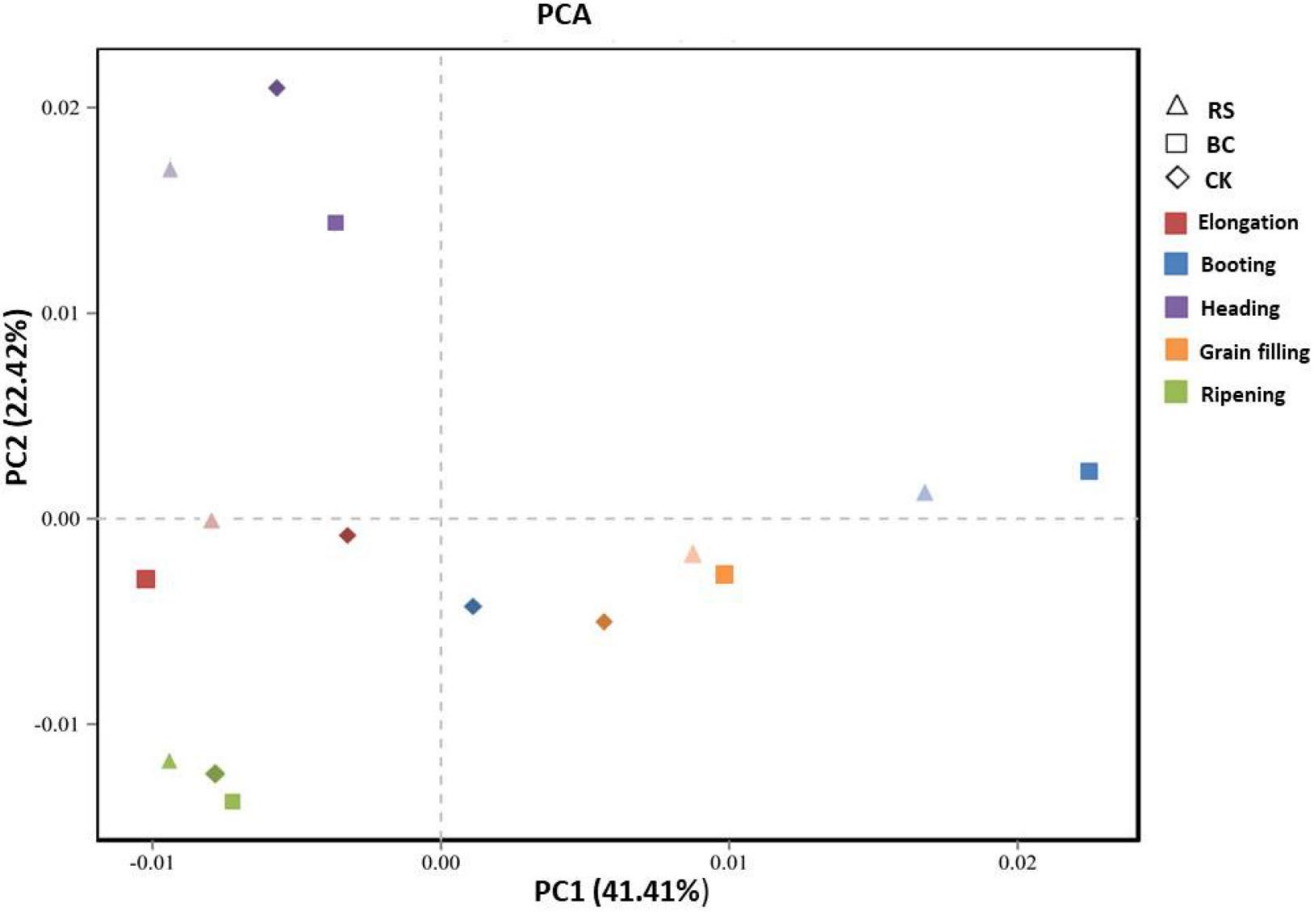
Principal component analysis of the bacterial genera in the rhizosphere soil samples. Treatments: *RS* rice straw applied at 9000 kg ha^−1^, *BC* biochar applied at 3150 kg ha^−1^, *CK* soil without straw/biochar application.

Over the course of rice growth, the composition of rhizosphere microbial communities changed significantly within each of the three treatment groups (Additional file: Figure S1). In all three treatment groups, *Chloroflexi* abundance was lower at the jointing, heading, and ripening stages of growth, whereas it was elevated during the booting and filling stages. *Proteobacteria* abundance trended downwards from the elongating to the booting stage, whereas it slowly trended upwards from the booting to the ripening stage in RS-treated (Additional file: Figure S1, RS) and BC-treated (Additional file: Figure S1, BC) samples. In contrast, *Proteobacteria* abundance in CK samples remained unchanged from the elongating to the booting stage, declined at the heading stage, and rose at the ripening stage (Additional file: Figure S1, CK). *Actinobacteria* abundance declined from the elongation to the booting stage, and rose slowly under all treatment conditions. A principal component analysis (PCA) was conducted to further clarify the relationships among microbial communities under these three treatment conditions during the five growth stages. This analysis was conducted using a Mothur calculation approach based upon relative bacterial abundance at the genus level, and the results were visualized using R v.3.0.2 (Fig. 3). In the resultant 2D plot, PC1 and PC2 respectively corresponded to 41.4% and 22.4% of the overall variation. Significant differences among the five growth stages were detected during this analysis, with bacterial communities during the elongation, booting, and grain filling stages being more similar than communities collected during the heading and ripening stages of growth. In addition, differences among treatment conditions were noted for samples collected at the elongation, booting, heading, and grain filling stages, whereas these samples appeared similar to one another at the ripening stage.

### RS and BC amendment alter the rhizosphere soil microbial community composition

**Figure 4:**
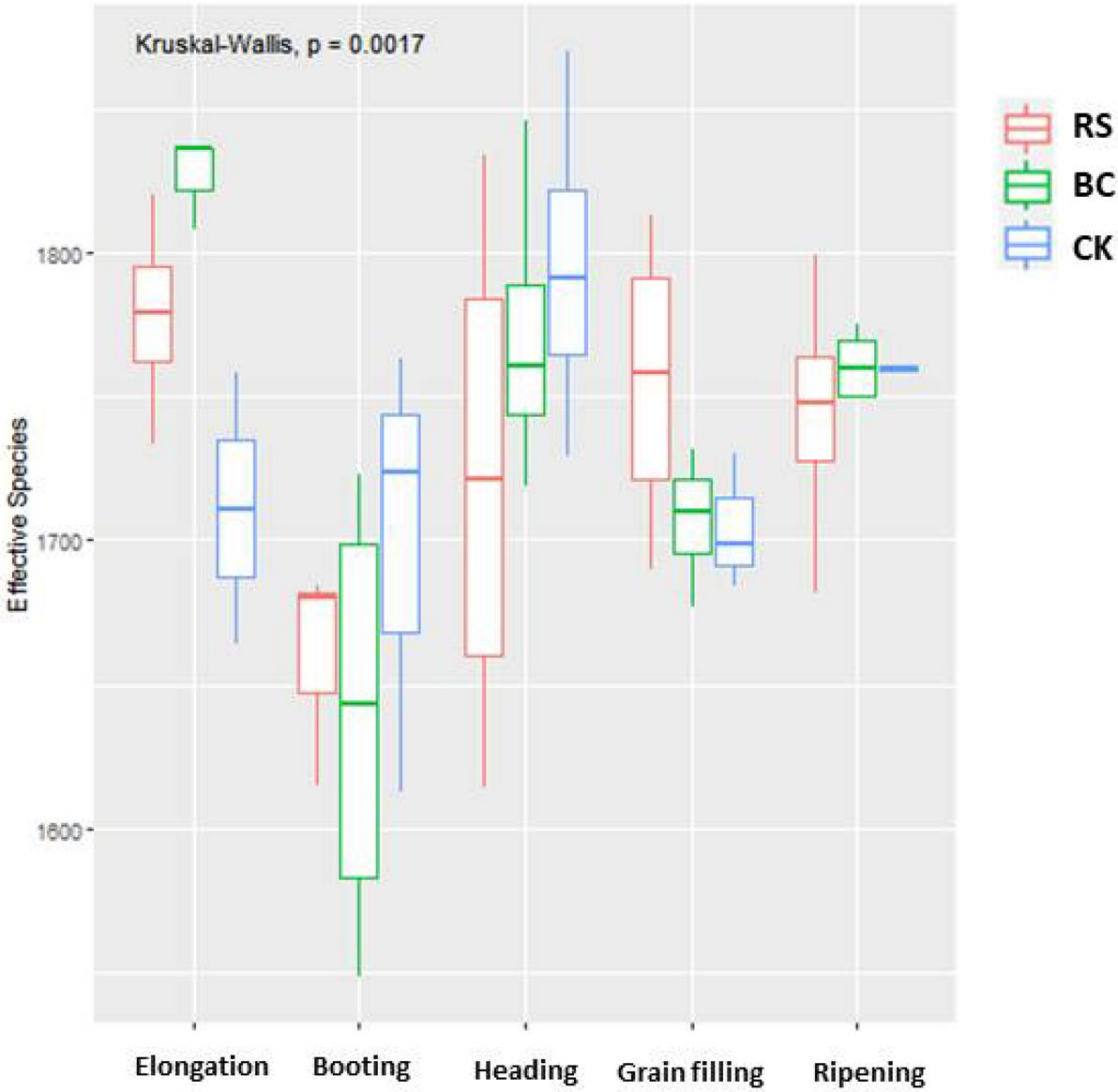
Alpha diversity indices for microbes in rhizosphere soil samples collected during different stages of rice growth. Treatments: *RS* rice straw applied at 9000 kg ha^−1^, *BC* biochar applied at 3150 kg ha^−1^, *CK* soil without straw/biochar application.

Significant differences in rhizosphere α-diversity values were detected among samples across the five rice growth stages (P = 0.0017, Kruskal–Wallis, Fig. 4). In elongation stage samples, α-diversity values were higher for both RS and BC samples relative to CK controls, whereas RS and CK samples exhibited higher α-diversity values than BC samples at the booting stage of growth. At the heading stage, BC and RS samples exhibited reduced α-diversity relative to CK samples, while BC samples presented with increased α-diversity relative to RS and CK samples at the grain filling stage, while differences between the BC and CK samples were not significant. No significant differences were detected among groups when comparing samples collected at the ripening stage.

### Relative root microbial abundance

**Figure 5:**
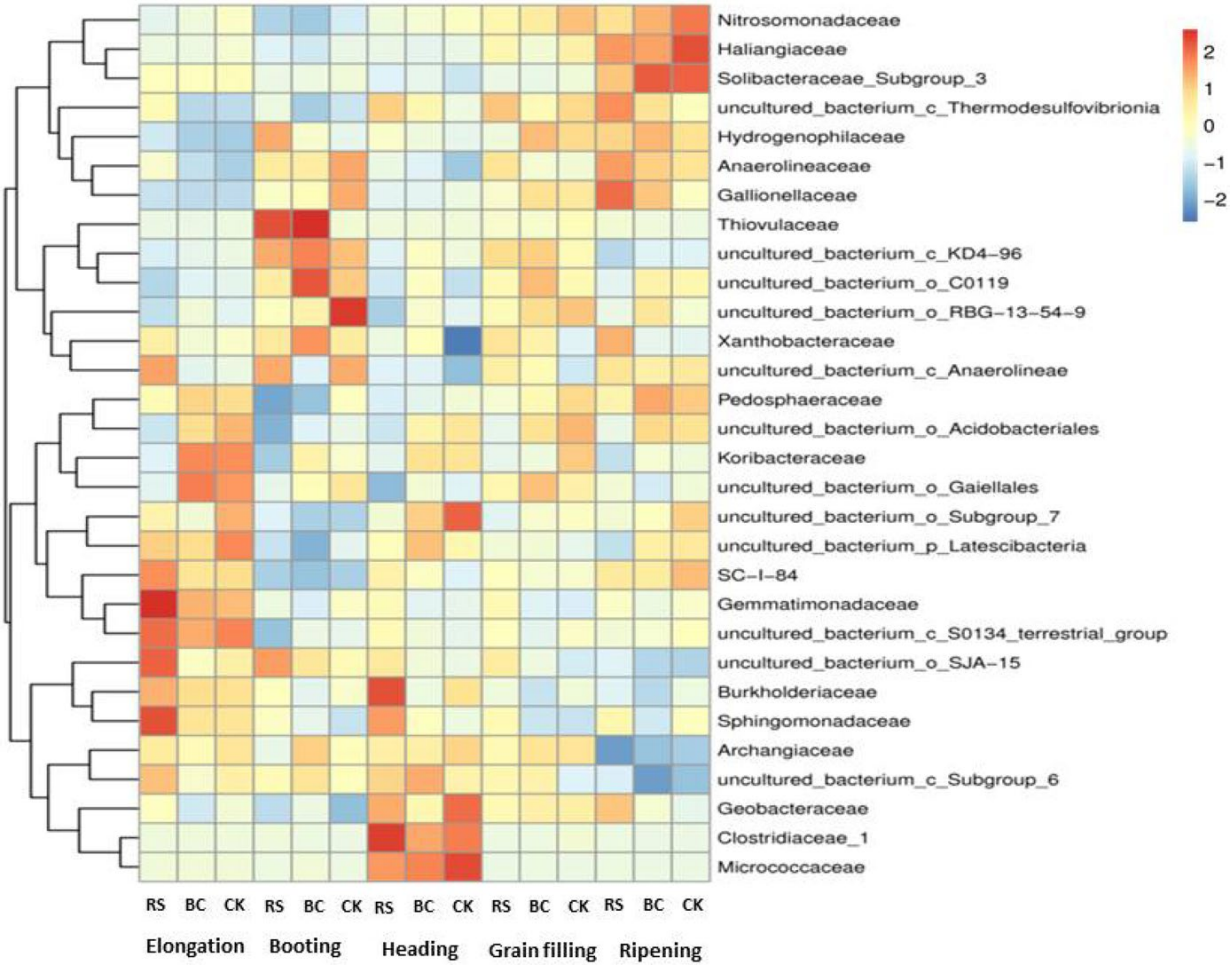
Relative abundance of top 30 genera in the treatment groups using R language (R v 3.1.1, https://developer-platform.biocloud.net/#/Microbial/taxonomySpecies/abundance_chart) to construct a species abundance heat map. Relative abundance is the correlation between species and treatments, with red representing a positive correlation and blue a negative correlation Treatments: *RS* rice straw applied at 9000 kg ha^−1^, *BC* biochar applied at 3150 kg ha^−1^, *CK* soil without straw/biochar application.

Differences were found in the relative abundance of the top 30 genera among different treatments (Fig. 5). At the elongation stage, the dominant bacterial families in RS-treated samples were *Gemmatimonadaceae* and *Sphingomonadaceae*, while no families were clearly dominant in BC or CK samples. At the booting stage, *Thiovulaceae* was the dominant family in RS-treated samples of rhizosphere soil, while *Thiovulaceae* and *uncultured* *bacterium-o-C0119* were dominant in BC-treated soil, and *Uncultured-bacterium-o-RBG-13-54-9* was dominant in the CK soil. At the heading stage, *Burkholderiaceae and Clostridiaceae-1* were dominant in the RS-treated soil, while *Uncultured-bacterium-o-Subgroup-7* and *Micrococcaceae* were dominant in the CK soil and no families were clearly dominant in BC-treated soil samples. No families were clearly dominant under any treatment conditions at the grain filling stage, while at the ripening stage *Haliangiaceae* was dominant in CK samples, whereas RS- and BC-treated soils did not exhibit any dominant bacterial families.

## Discussion

Prior analyses have shown that crop rhizosphere microbial community composition and diversity vary depending on the types of plant residues applied to a given field, but these studies were conducted using upland crops or under greenhouse conditions^14,18,19^. In the present study, we focused on paddy soil rhizosphere microbes associated with rice plants, and assessed the Chao1 and Shannon diversity indices for these communities following RS (9000 kg ha^−1^), BC (3150 kg ha^−1^), or control treatment during five stages of growth, enabling us to detect significant variations in soil microbiome diversity (Table 1). However, the OTUs in these samples did not differ significantly across all treatment conditions (Fig. 1), as these OTUs did not change in total quantity following RS or BC application relative to CK. RS and BC amendment only altered the relative abundance of these root-associated microbes at the phyla level, consistent with the results previously published by Novak^6^ and Zhao^20^, who determined that the addition of RS and BC can increase available soil organic C and thereby provide a niche for soil microbial growth.

*Chloroflexi, Proteobacteria,* and *Acidobacteria* were the dominant phyla detected during all five stages of rice growth in the present study (Fig. 2), in line with prior research^21–23^. We found that *Proteobacteria* were the most abundant phyla in the RS-treated samples during the different stages of growth (Additional file: Figure S1, RS). This is consistent with reports that *Proteobacteria* are typically the dominant microbial phylum found in soil samples^24,25^. *Proteobacteria* can readily grow in nutrient-rich soil, and RS decomposition can facilitate significant nutrient release into soil ecosystem, supporting the growth of large numbers of these bacteria surrounding the root system. *Chloroflexi* was the most abundant phylum in the RS and BC samples across all analyzed rice growth stages (Additional file: Figure S1, BC, CK). *Chloroflexi* are anaerobic bacteria, and many microbes can grow in the rhizosphere under anaerobic conditions. The relative abundance of *Chloroflexi* in the analyzed samples varied with growth stage in an “M”-shaped pattern, potentially due to cultivation-related conditions. During the booting and grain filling stages of rice growth, paddy soil is saturated with water, whereas this water is not present during other growth stages. BC application increased relative rhizosphere soil *Chloroflexi, Proteobacteria,* and *Acidobacteria* abundance, likely because the BC provided high levels of C and an environmental niche compatible with the growth of these root-associated microbes owing to its stable, porous structure. Other groups have previously reported that BC addition can stimulate soil bacteria growth, with root-associated microbiota changing accordingly over the course of crop growth^19,26^, in line with our findings. High bacterial diversity and richness are typically associated with the proliferation of microbes capable of utilizing more diverse sources of carbon^27^. RS and BC application increased the relative *Proteobacteria, Actinobacteria*, and *Gemmatimonadetes* abundance in rhizosphere soil samples, consistent with the ability of these microbes to flourish in nutrient-rich settings^28^. Most eutrophication *Proteobacteria* in the RS- and BC-treated soil samples were present at higher levels than in CK samples at multiple growth stages (Fig. 2), suggesting that nutrient levels remained high in the amended rhizosphere soil throughout the growth period. *Nitrospirae* levels have been shown to rise following tillering and to remain at high levels during the rice reproductive stage^29,30^. Significant rises in *Nitrospirae* abundance in RS- and BC-treated rhizosphere soils (Fig. 2) can markedly enhance rice nitrate uptake. Overall, rhizosphere soil bacterial diversity and richness can be impacted by a range of variables including soil composition, cultivation methods, and management practices^31^. Analyzing the diversity of soil microbial communities can offer insight regarding the stability and fertility of the soil ecosystem, with *Proteobacteria* flourishing in nutrient-rich environments, while oligotrophic *Actinobacteria* tend to dominate in nutrient-poor environments wherein these slow-growing microbes can degrade more recalcitrant forms of C^32^. Consistent with the beneficial effects of RS application, we detected high levels of *Proteobacteria* in the RS-treated rhizosphere soil, indicating that C levels were abundant throughout the rice growth cycle, while the presence of *Actinobacteria* in BC-treated soils may indicate an environment that was somewhat more nutrient-poor.

Genus-level PCA results indicated that both RS and BC amendments significantly impacted soil microbial community composition from the elongation stage to the grain filling stage, although these differences were no longer evident at the ripening stage (Fig. 3). Prior reports have also shown that RS application can significantly alter soil microbial α-diversity^33^. Indeed, we observed significant differences in rhizosphere α-diversity measurements in samples from the RS and BC treatment groups relative to CK control samples (Fig. 4). *Gemmatimonadaceae, Sphingomonadaceae, Thiovulaceae, Burkholderiaceae,* and *Clostridiaceae-1* were the dominant families of microbes in RS-treated samples, whereas *Thiovulaceae* and *uncultured-bacterium-o-C0119* were dominant in BC-treated samples (Fig. 5)*.* RS application thus significantly altered the soil microbial community structure. Other studies have shown that levels and qualities of compounds released from crop straw can contribute to consequent changes in soil microbial communities^11^.

Straw and biochar harbor large quantities of C, which serves as the main energy source for soil microorganisms^39^. *Chloroflexi, Acidobacteria, Actinobacteria,* and *Verrucomicrobia* levels were higher in BC samples relative to RS samples (Fig. 2). *Chloroflexi* are anaerobic bacteria, and the anaerobic conditions that arise during rice growth normalize and regulate soil bacterial community composition based on their oxygen requirements, resulting in stable bacterial communities in paddy soils^40^. *Acidobacteria* are acidophilic bacteria that play important roles in ecosystems, being primarily responsible for the decomposition of plant residue in the soil. Relative to straw application, BC input was associated with the increased abundance and diversity of these phyla under the water layer, potentially because the pH value of the soil solution may be changed due to alkalinity of the BC, and maybe because these bacteria are able to inhabit the microporous structure of the BC. BC application may also alter other environmental factors including soil moisture, temperature, and pH.

BC amendment was associated with a significant decrease in *Proteobacteria, Gemmatimonadetes, Nitrospirae,* and *Firmicutes* abundance relative to CK treatment during all growth stages in the present study (Fig. 2). *Gemmatimonas* were an abundant bacterial phyla associated with the RS treatment, potentially because *Gemmatimonas* use the RS as the sole source of available C^41^. Indeed, *Gemmatimonas* have been shown to reduce the metabolic products of cellulose^41^, thereby indirectly facilitating cellulose degradation. *Nitrospirae* species play pivotal roles in nitrification by oxidizing nitrite to nitrate^29^. The enrichment of *Nitrospirae* may be due to root environmental changes during rice growth such as pH changes or root exudates. It is also possible that these microbes were actively recruited by rice to facilitate nitrate assimilation, potentially providing advantages with respect to nutrient uptake for rice cultivars during the elongating and booting stages. In this study, the relative abundance of Proteobacteria and Verrucomicrobia species was related to soil nutrient availability. We thus speculated that RS treatment may stimulate the relative proliferation of copiotrophic bacteria owing to the increased soil nutrient availability associated with RS decomposition.

At present, the application of RS to fields is the most common amendment method. In addition to contain large quantities of nutrients and trace elements, crop straw application can also increase the levels of soil organic matter, improve soil fertility, and significantly improve crop yields^42^. However, direct straw application is associated with issues including low temperatures, slow decomposition, and greenhouse gas emissions in northeast China^43^. BC is applied to fields in an innovative form of straw application. BC primarily affects the structure and function of microbial communities by maintaining carbon stability, improving soil quality, enhancing water retention, and changing pH^44^.

## Conclusion

Our results showed that the applications of RS and BC for rice production did not increase the number of observed OTUs, type of dominant phylum, family or genera variety in the rhizosphere soil bacterial community. On the other hand, RS and BC addition improved rhizosphere soil bacterial diversity and richness in phyla (*Chloroflexi, Proteobacteria,* and *Acidobacteria*) and clearly impacted the distribution of dominant family such as *Gemmatimonadaceae, Sphingomonadaceae, Thiovulaceae, Thiovulaceae and uncultured-bacterium-o-C0119*. Principal component analysis showed that the rhizosphere soil bacteria community differed significantly among the RS, BC and CK at all five stages of rice growth. RS and BC amendments have positive effects on soil fertility and productivity, helping farmers to reduce costs associated with straw disposal while increasing grain yields and improving production efficiency. Such amendment may also help improve soil productivity and sustainability in intensive agricultural systems. In comparison with synthetic fertilizers alone, the application of the RS and BC can improve soil microbial diversity and richness for rice production in Northeast China.

## Materials and methods

### Experimental design

The present study was conducted at Liaoning Rice Research Institute (41°47′ N, 123°34′ E, altitude: 40.5 m) in Shenyang, Liaoning Province, China. Field experiments were initiated in 2019 to assess the impact of RS and BC amendment on soil bacterial community structure and biological traits. The soil at the study site is of a clayey loam type. At the start of the experiment, the soil exhibited a pH (H_2_O) of 5.2,19.2 g kg^−1^ organic matter, 1.29 g kg^−1^ total N, 1.27 g kg^−1^ total P, 23.7 g kg^−1^ total K, 109.0 mg kg^−1^ available N, 24.5 mg kg^−1^ available P, and 45.0 mg kg^−1^ available K. The study site is located in a region with a semi-humid temperate and monsoonal type climate, with annual average temperature and precipitation of 8.2 °C and 550 mm, respectively.

The field experiment was designed with three treatments and four replicates per treatment in a completely randomized design, with each plot being 360 m^2^ in area. The treatments were as follows:

1. Rice straw (RS) was applied at 9000 kg ha^−1^. RS was prepared by chopping dried straw and passing it through a 10 mm sieve. The resultant RS contained respective C and N levels of 354.8 g kg^−1^ and 6.8 g kg^−1^.
2. Biochar (BC) was applied at 3150 kg ha^−1^. BC was prepared by subjecting RS to pyrolysis for 1 h under low-oxygen conditions at 450 °C (Jin and Fu Agriculture Development Co., Ltd), as this is comparable to the commercial process used during traditional furnace-based corn cob carbonization (Chinese patent ZL 2007 10086505.4). Using this approach, roughly 35% of the RS was converted into BC in the form of granular particles that were 2 mm in diameter. The resultant BC contained 660.4 g kg^−1^ of C and 7.9 g kg^−1^ of N, and exhibited a pH of 8.6 (1:2.5 H_2_O).
3. Control (CK) treatment without any soil amendment.

RS and BC were applied to experimental sites prior to rice transplanting in April 2019. These materials were incorporated into the field by hand using a rake, after which all plots were mechanically tilled to a uniform 0.15 m depth. The soil was additionally amended with calcium superphosphate and potassium chloride (615 kg P_2_O_5_ ha^−1^ and 200 kg K_2_O ha^−1^), with additional urea being applied at the mid-tillering (270 kg ha^−1^) and panicle initiation (270 kg ha^−1^) stages of rice growth. P and K fertilizers were evenly applied as basal fertilizers on the soil surface and were promptly incorporated in the plow layer (0–10 cm) using a hand rake prior to transplanting.

The rice cultivar ‘Liaoxing 21’ was cultivated in 2019. The variety was widely used in a large northern part of area in Liaoning Province, and conventionally grown by local farmers. Rice seeds were sown in a nursery bed on April 19, 2019. Seedlings were transplanted to the paddy fields on May 26, 2019 and then harvested on October 20, 2019. Water levels, weed growth, insects, and diseases were managed as appropriate to prevent yield losses.

### Rhizosphere soil collection

Soil samples were collected at the elongation, booting, heading, grain filling, and ripening stages of rice growth. At these times, plants and soil samples were collected from each plot, and five soil cores were pooled together to yield a composite sample. Three replicate samples were collected per treatment. Roots were removed from the soil and shaken to remove excessive soil until ~ 1 mm of soil remained attached. This remaining soil was then obtained by placing roots in a flask containing 50 mL of sterile PBS. The solution was then vigorously stirred with sterile forceps to detach the rhizosphere soil, which was transferred to a 50 mL tube and frozen at − 80 °C prior to DNA extraction.

### 16S rDNA sequencing and analysis

DNA was isolated from 0.5 g of soil with a Fast DNA SPIN Kit for Soil (Q-BIOgene, CA, USA) based on provided directions. DNA quality and quantity were assessed using an automated microplate reader (SynergyHTX, Gene Company Limited). The bacterial 16SrRNA V3-V4 region was then amplified with the 338F (5′-ACTCCTACGGGAGGCAGCA-3′) and 806R (5′-ACTCCTACGGGAGGCAGCA-3′) primers. A thermocycler (ABI GeneAmp 9700) was used for PCR amplification (25 or 10 cycles), with each reaction containing a total 10 μl or 20 μl volume. Successful amplification was confirmed via 1% agarose gel electrophoresis. After electrophoretic quantification using the ImageJ program, PCR products of all samples were combined together based on quality ratio values at a 1:1 ratio. Samples were purified using an OMEGA DNA purification column, and were mixed at equimolar ratios prior to sequencing with an Illumina HiSeq PE150 instrument (Illumina, USA) by Biomarker Technologies Co, LTD. Raw sequencing data were then merged with FLASH^34^, mass filtered^35^, and chimeric sequences removed to yield high-quality tagged sequences^36^. These sequences, in turn, were clustered at a 97% similarity level^37^.

### Statistical analyses

Mothur was used to calculate OTU richness (ACE, Chao1), Shannon, and Simpson diversity indices^38^. Significant differences in microbial community composition among samples were identified via a permutational multivariate analysis of variance (PERMANOVA) approach. Microsoft Excel 2010 was used to graph the resultant data. Mothur was additionally used to compute principal component coordinates, while R (v 3.0.2) was used to construct a PCA analysis diagram. R language (R v 3.1.1, https://developer-platform.biocloud.net/#/Microbial/taxonomySpecies/abundance_chart) was used to construct a species abundance heat map.

### Ethical standards

This project and the experiments were conducted in strict compliance with the IUCN Policy Statement on Research Involving Species at Risk of Extinction and the Convention on the Trade in Endangered Species of Wild Fauna and Flora. In our study that all plant research was carried out in accordance with national, international or institutional guidelines.

## Supplementary Information

Supplementary Figure S1.
