# Supplementary material for: Soil bacterial community as impacted by addition of rice straw and biochar
Source: Sci Rep. 2021 Nov 12;11:22185. doi: 10.1038/s41598-021-99001-9 (PMC8589988; doi:10.1038/s41598-021-99001-9)
Supplement: Supplementary file 1 — Supplementary Figure S1. [file 41598_2021_99001_MOESM1_ESM.docx]

**Soil bacterial community as impacted by addition of rice straw and biochar**

**Zhiqiang Tang^1,2^, Liying Zhang^2^, Na He^2^, Diankai Gong^2^, Hong Gao^2^, Zuobin Ma^2^, Liang Fu^2^, Mingzhu Zhao^2^, Hui Wang^2^, Changhua Wang^2^, Wenjing Zheng^2^, Wenzhong Zhang^1*^**

**Affiliation: ^1^** **Shenyang Agricultural University, Shenyang, China.**

**^2^ Liaoning Rice Research Institute, Shenyang, China.**


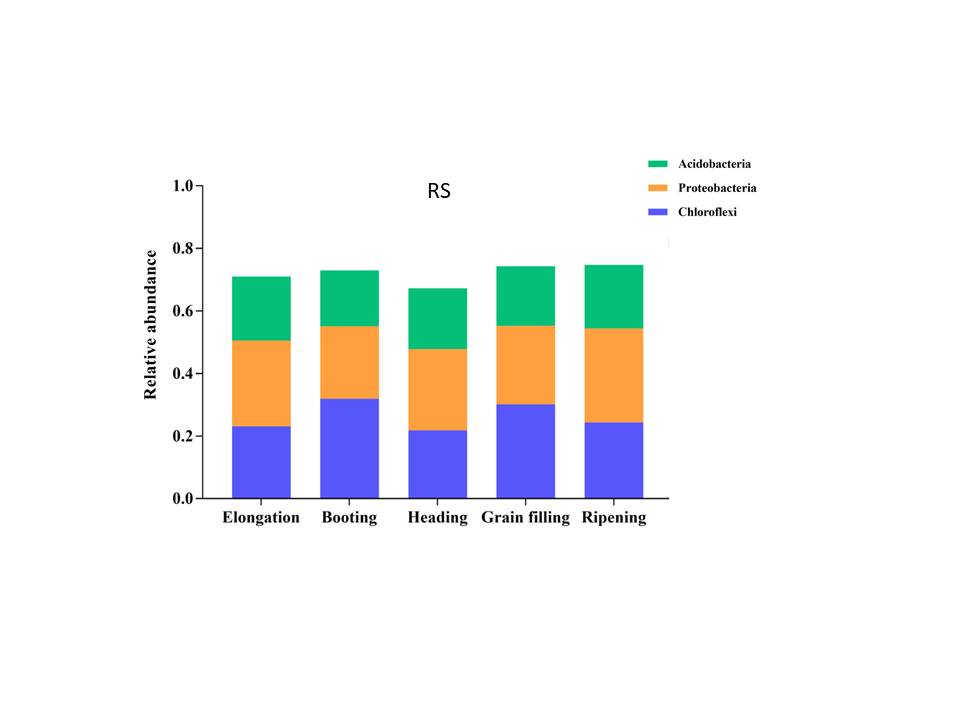


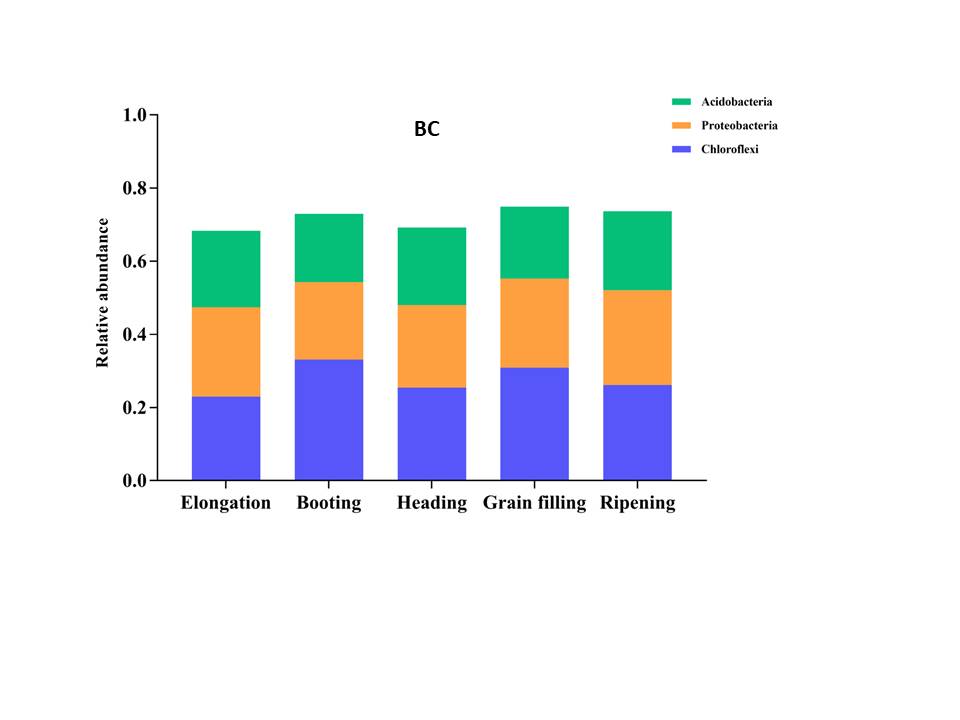


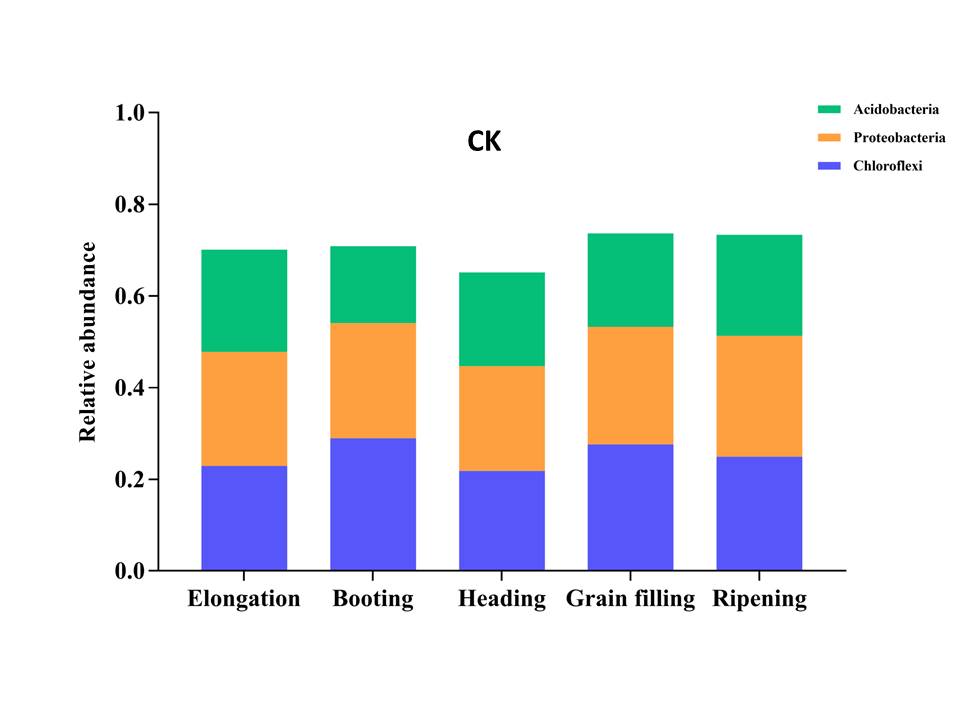


Figure S1. The relative abundance of the phylum Chloroflexi, Proteobacteria and Acidobacteria in the rhizosphere at all growth stages. Treatments: RS = Rice straw applied at 9000 kg ha^−1^; BC = Biochar applied at 3150 kg ha^−1^; CK = Soil without straw/biochar application.
